# Supplementary material for: AAV9:PKP2 improves heart function and survival in a Pkp2-deficient mouse model of arrhythmogenic right ventricular cardiomyopathy
Source: Commun Med (Lond). 2024 Mar 18;4:38. doi: 10.1038/s43856-024-00450-w (PMC10948840; doi:10.1038/s43856-024-00450-w)
Supplement: Supplementary file 2 — Supplementary Information [file 43856_2024_450_MOESM2_ESM.pdf]

PKP2 Gene Therapy Improves Heart Function and Reduces Mortality in a *Pkp2*-deficient Mouse Model of Arrhythmogenic Right Ventricular Cardiomyopathy

Iris Wu[1,2*], Aliya Zeng[1*], Amara Greer-Short[1***], J. Alex Aycinena[1], Anley E. Tefera[1], Reva Shenwai[1], Farshad Farshidfar[1], Melissa Van Pell[1], Emma Xu[1], Chris Reid[1], Neshel Rodriguez[1], Beatriz Lim[1], Tae Won Chung[1], Joseph Woods[1], Aquilla Scott[1], Samantha Jones[1], Cristina Dee-Hoskins[1], Carolina G. Gutierrez[1], Jessie Madariaga[1], Kevin Robinson[1], Yolanda Hatter[1], Renee Butler[1], Stephanie Steltzer[1], Jaclyn Ho[1], James R. Priest[1], Xiaomei Song[1], Frank Jing[1], Kristina Green[1], Kathryn N. Ivey[1], Timothy Hoey[1], Jin Yang[1**], Zhihong Jane Yang[1**,***]

[1] Tenaya Therapeutics, South San Francisco, CA 94080, USA

[2] Present address: University of Michigan, Department of Molecular and Integrative Physiology, Ann Arbor, MI 48109-5622, USA

* These authors contributed equally.

**These authors jointly supervised this work.

*** Corresponding authors: jane.yang@tenayathera.com; agreer-short@tenayathera.com

Supplementary Figures and Table:

1) Supplementary Figure 1: PKP2 silencing led to reduction in protein expression of DSP, JUP, DES, and MyBPC3 in response to reduced PKP2 protein.
2) Supplementary Figure 2: Western blot analysis showed that the second generation of AAV:hPKP2 is expressed in iPSC-CMs in a dose-dependent fashion by applying viruses at different multiplicity of infection (MOI)
3) Supplementary Figure 3: Western blots of Pkp2-cKO mouse hearts showed reduction of desmosome proteins PKP2, DSP, JUP and gap junction protein Cx43.
4) Supplementary Figure 4: Enlarged EKG traces for main Figure 4b.
5) Supplementary Figure 5: TN-401 and AAV9:mPkp2 preserved ejection fraction and right ventricle size, reduced arrhythmia scores, and improved lifespan relative to vehicle-treated *Pkp2-cKO* Animals.
6) Supplementary Figure 6: AAV9:mPkp2 showed dose-dependent efficacy in preserving ejection fraction and right ventricle size, reducing arrhythmia scores, and improved lifespan relative to vehicle-treated *Pkp2-cKO* Animals.
7) Supplementary Figure 7: TN-401 showed dose-dependent expression of human *PKP2* transgene protein and dose-dependent restoration of desmosome proteins.
8) Supplementary Figure 8: TN-401 showed dose-dependent expression of human *PKP2* transgene protein.
9) Supplementary Table 1: Arrhythmia grade chart

Supplementary Figure 1: PKP2 silencing led to reduction in protein expression of DSP, JUP, DES, and MyBPC3 in response to reduced PKP2 protein.

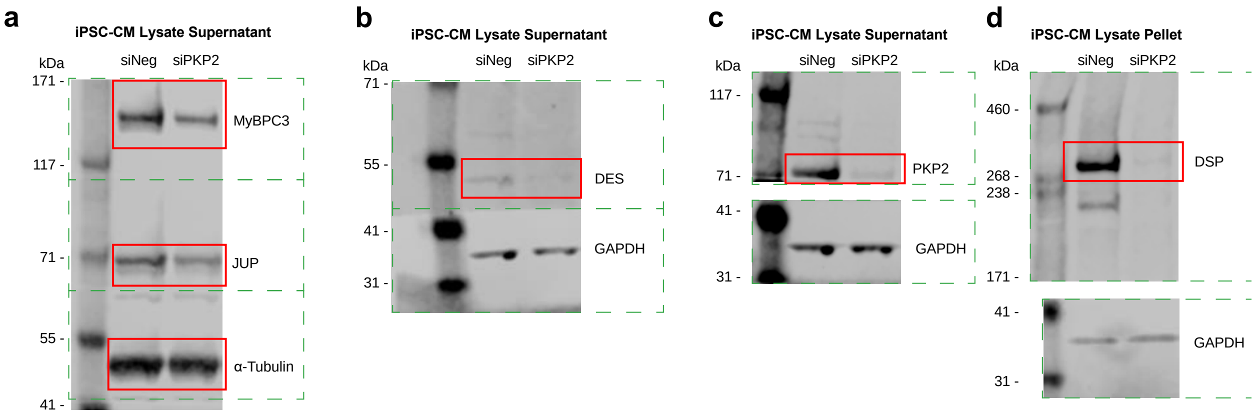

Western blots for main Figure1b. **a, b, and c**, After silencing wildtype iPSC-CMs with siPKP2 for eight days, MyBPC3, JUP, DES and PKP2 protein expression was assayed using the supernatant fraction of lysates. A control siRNA (siNeg) was used as a negative control. GAPDH or α-Tubulin was probed as a loading control. **d**, DSP protein expression was assayed using the pellet fraction of lysates. Green boxes represent where blots are cut into segments to probe for proteins of interest. Red boxes highlight the bands presented in the main text.

Supplementary Figure 2: Western blot analysis showed that the second generation of AAV:hPKP2 is expressed in iPSC-CMs in a dose-dependent fashion by applying viruses at different multiplicity of infection (MOI)

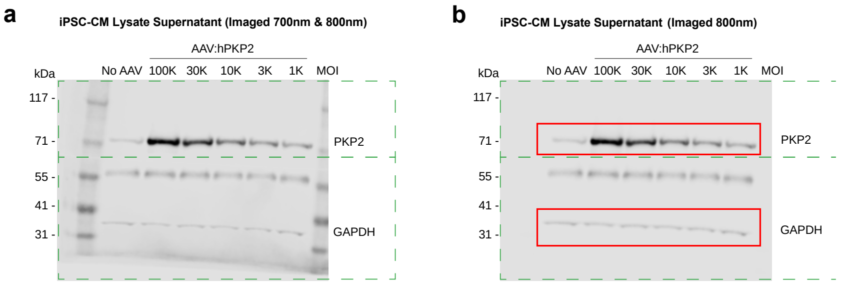

Western blots for main Figure2b. **a,** Blot imaging at 700nm is included to show the ladder. **b,** Expression of PKP2 protein in wildtype iPSC-CMs was assayed after treatment with increasing MOIs of AAV:hPKP2. GAPDH is probed as a loading control. Green boxes represent where blots are cut into segments to probe for proteins of interest. Red boxes highlight the bands presented in the main text.

Supplementary Figure 3: Western blots of *Pkp2-cKO* mouse hearts showed reduction of desmosome proteins PKP2, DSP, JUP and gap junction protein Cx43.

Supplementary Figure 3a

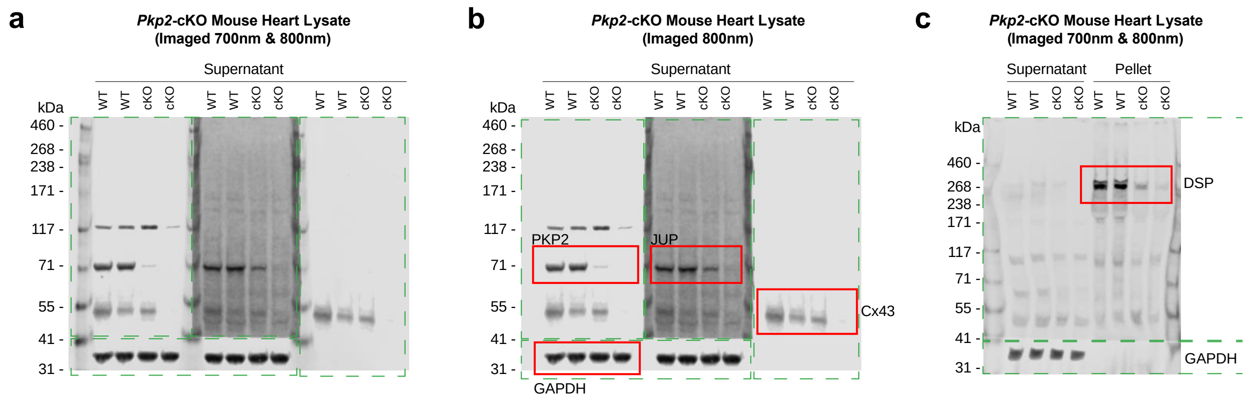

Western blots for main Figure3a. **a,** Blot imaging at 700nm is included to show the ladder. **b,** *Pkp2-cKO* reduced levels of JUP and Cx43 in response to reduced PKP2 expression as seen in the supernatant fraction of *Pkp2-cKO* mouse heart lysates. GAPDH is probed as a loading control. **c**, DSP expression is also reduced as seen in the pellet fraction of *Pkp2-cKO* mouse heart lysates. Green boxes represent where blots are cut into segments to probe for proteins of interest. Red boxes highlight the bands presented in the main text.

Supplementary Figure 4: Enlarged EKG traces for main Figure 4b.

a

WT, HBSS

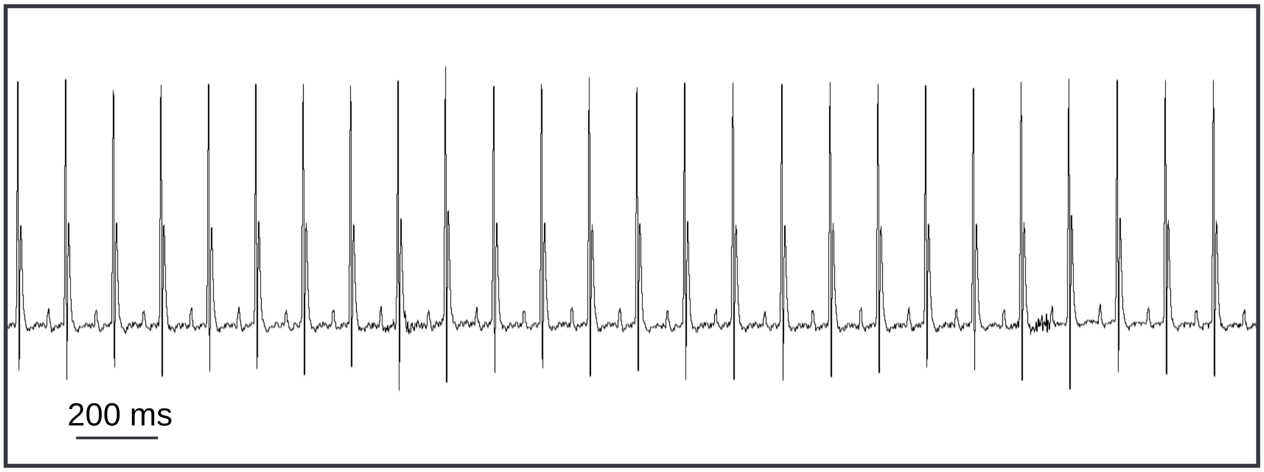

b

WT, HBSS, amplified trace

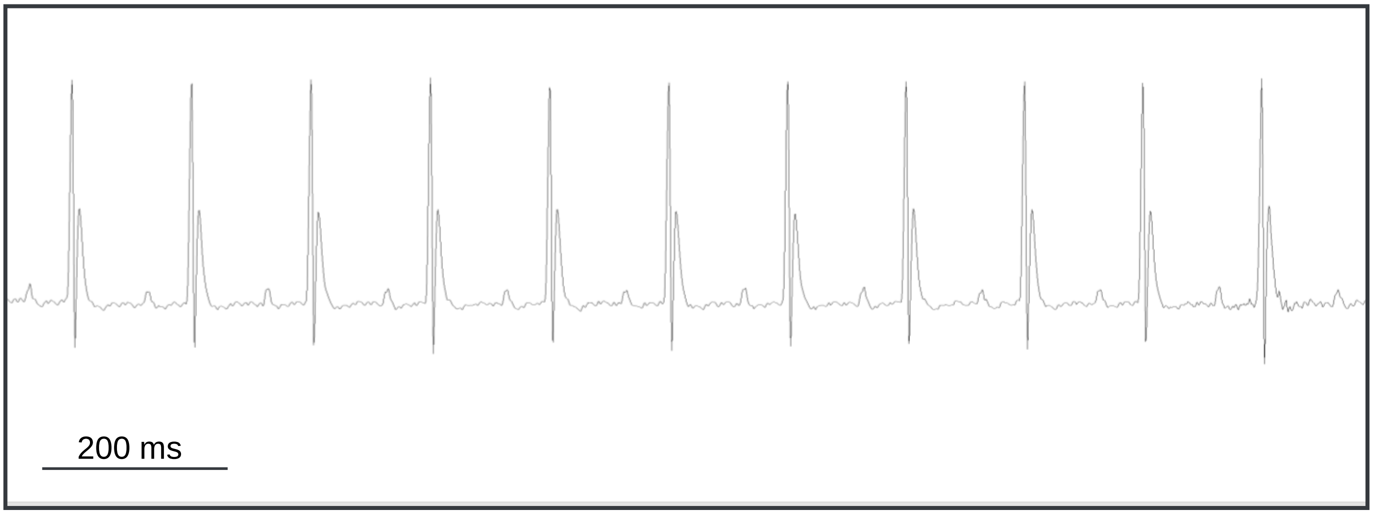

c

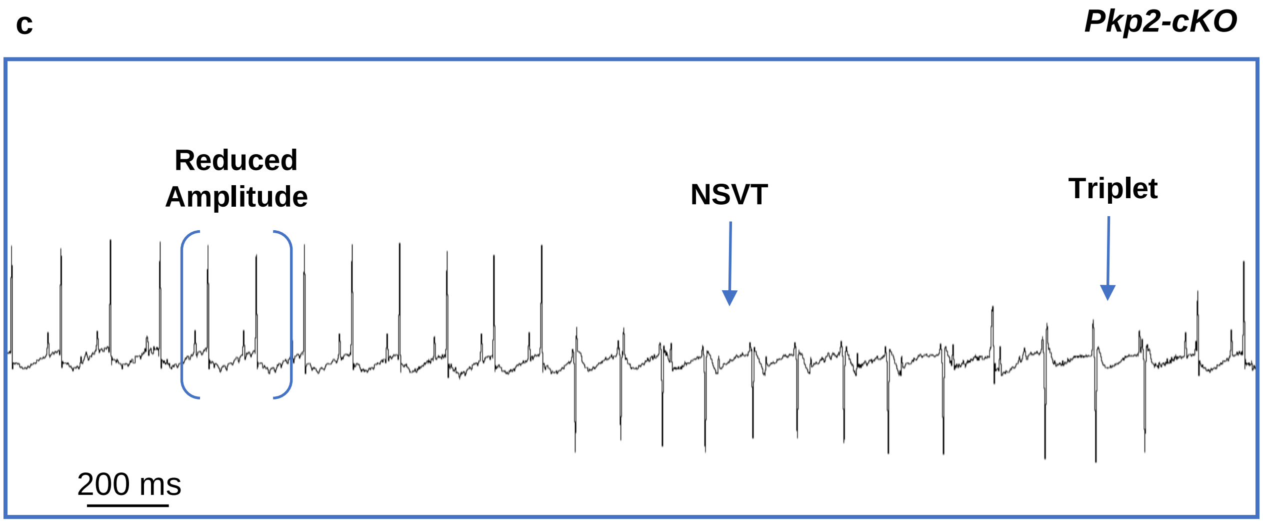
Pkp2-cKO
Reduced
Amplitude
NSVT
Triplet
200 ms

d

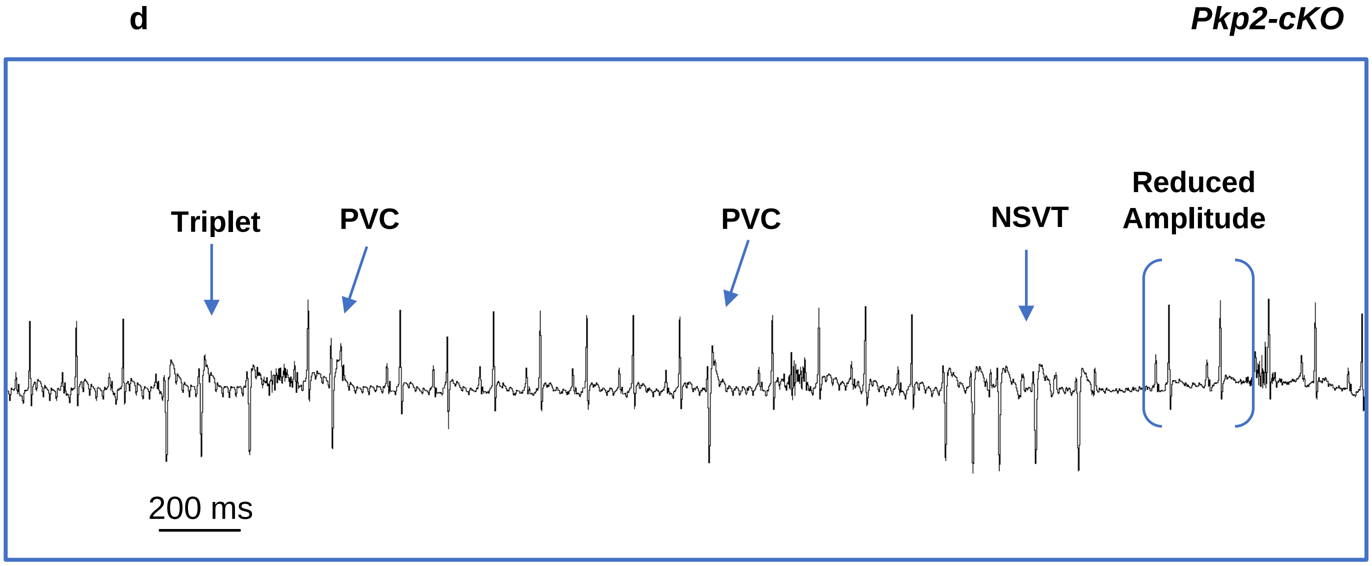
Pkp2-cKO
Triplet
PVC
PVC
NSVT
Reduced
Amplitude
200 ms

e

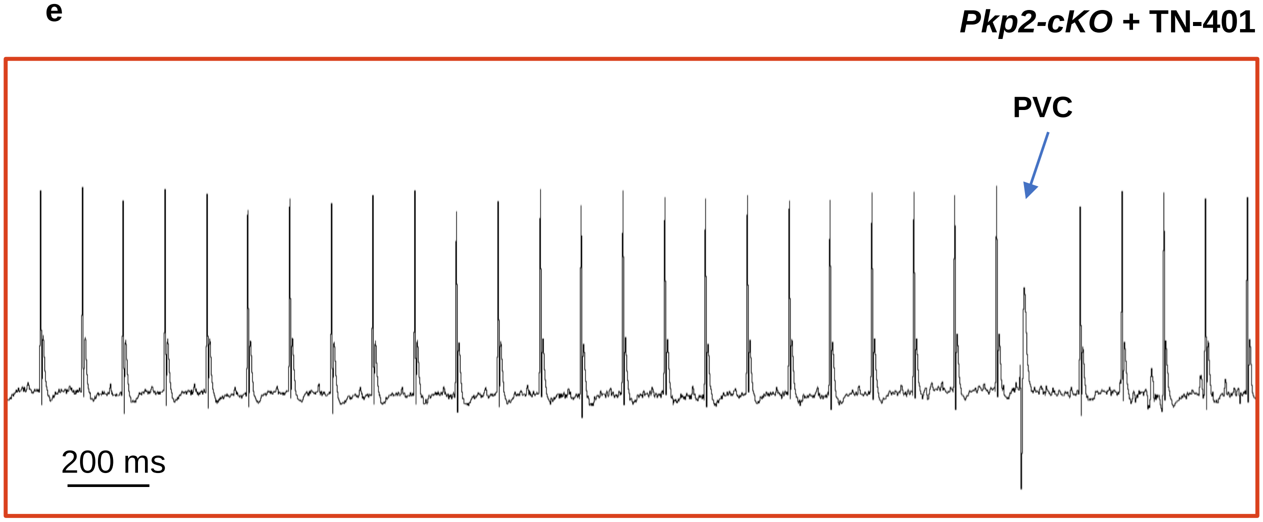

f

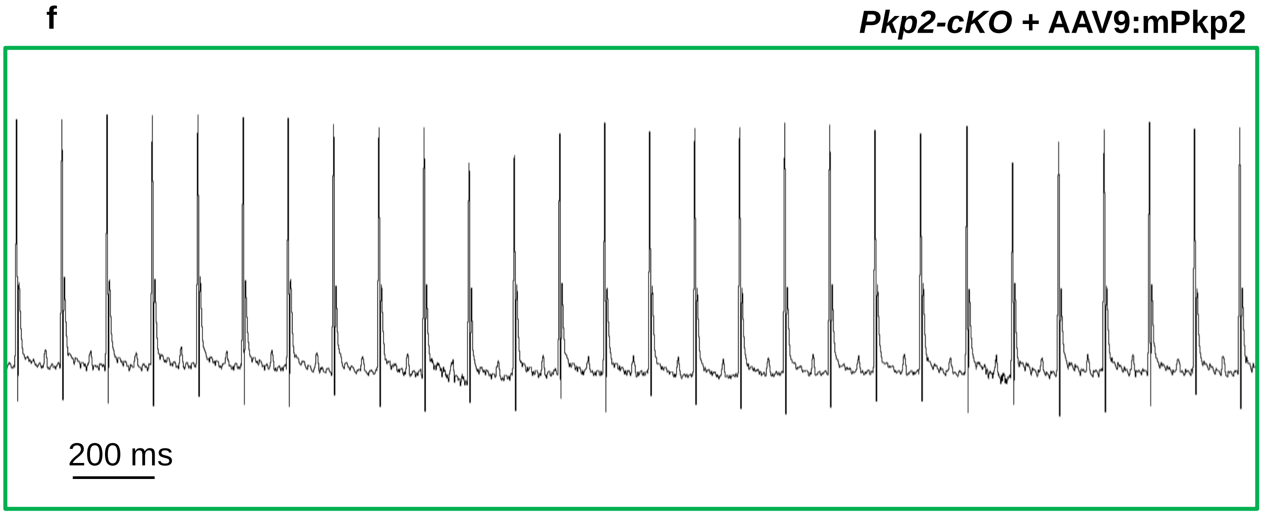

Enlarged EKG traces from main Figure 4b. **a,** EKG trace example from WT animal. As seen, the animal is in normal sinus rhythm. **b,** the amplified EKG trace from the WT animal so that PR, QRS, and QT intervals can be more clearly seen. **c and d,** EKG trace examples from two *Pkp2-cKO* animals. Animals had high frequency of PVCs and/or NSVT. **e,** EKG trace example from *Pkp2-cKO* animal with TN-401 treatment. **f,** EKG trace example from *Pkp2-cKO* animal with AAV9:mPkp2 treatment.

Supplementary Figure 5: TN-401 and AAV9:mPkp2 preserved ejection fraction and right ventricle size, reduced arrhythmia scores, and improved lifespan relative to vehicle-treated *Pkp2-cKO* Animals.

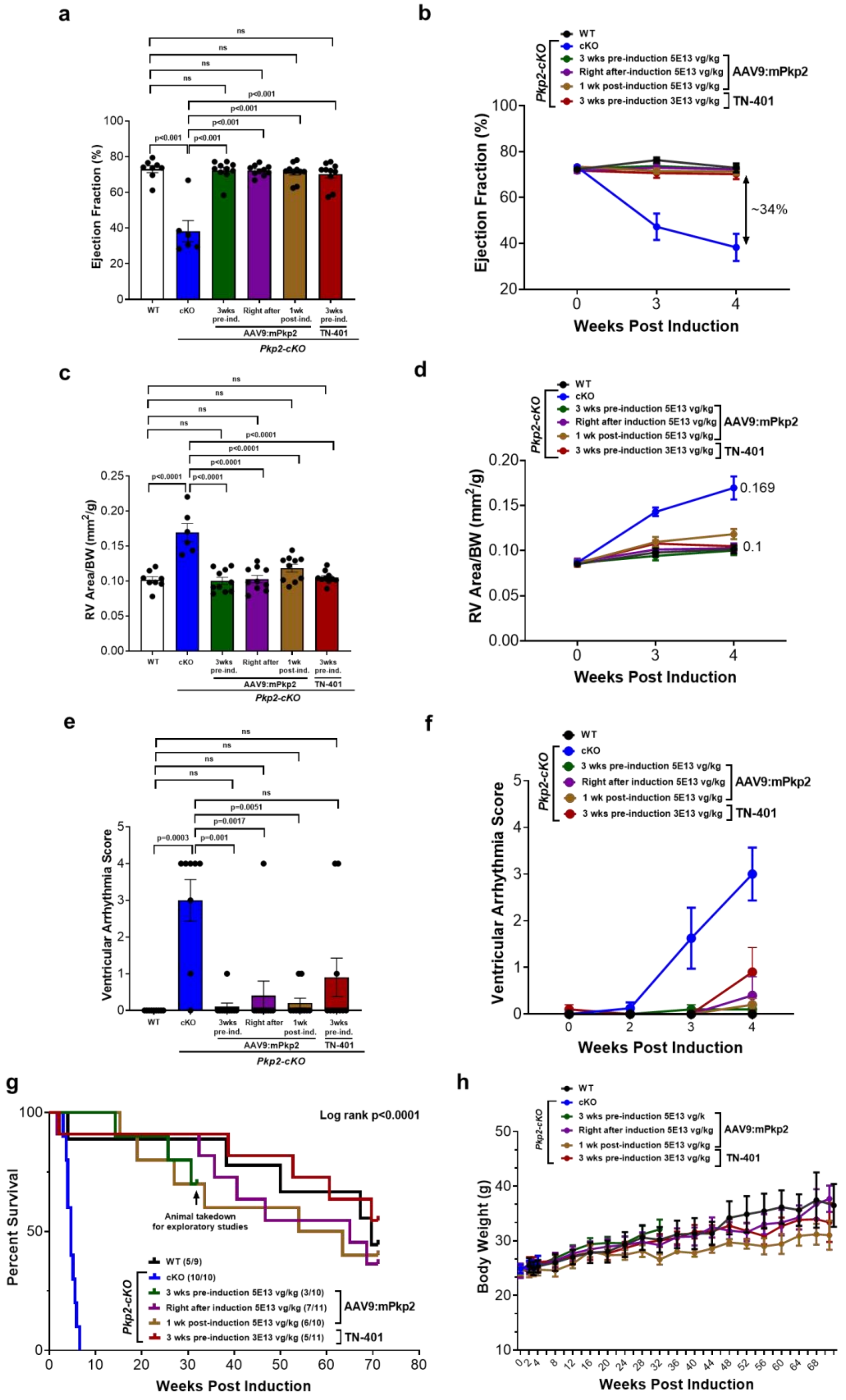

AAV9 was injected at three weeks before, right after, or 1 weeks after induction of *Pkp2* gene deletion. TN-401 was dosed at 3E13 vector genomes per kilogram bodyweight (vg/kg) and AAV9:mPkp2 at 5E13 vg/kg. Echocardiograph (Echo) and electrocardiogram (EKG) data were collected at week 3 and week 4 post gene deletion. **a,** EF% and **b,** EF% progression at 4 weeks post-induction. RV chamber dilation was measured by RV area normalized to body weight. **c,** RV/BW and **d,** RV/BW progression at 4 weeks post-induction. EF% and RV dilation in response to AAV9:PKP2 treatment was statistically evaluated with ordinary One-Way ANOVA (Tukey's post-hoc test). Animals that were euthanized or died prior to the last echo were excluded from the EF progression and LV Mass graphs. Incidence of ventricular arrhythmia was quantified during 30 minutes of recording of anesthetized animals. The frequency and severity of the spontaneous arrhythmia were recorded and categorized based on the grading chart in Supplementary Table 1. **e,** Ventricular arrhythmia score distribution of individual animals at 4 weeks post tamoxifen induction and **f,** ventricular arrhythmia score progression. Statistical significance in response to AAV9 treatment was evaluated using Kruskal-Wallis test with Dunn's correction. **g,** Kaplan–Meier survival curve showed that both TN-401 and AAV9:mPkp2 treatment extended median lifespan ≥ 58 weeks vs 4.7 weeks observed in the vehicle treated *Pkp2-cKO* animals. *Pkp2-cKO* animals treated 3 weeks before induction with AAV9:mPkp2 were euthanized at 32 weeks for exploratory *ex-vivo* studies. Numbers in paratheses showed dead vs live animals by the time of takedown. **h,** Animals were weighed weekly from start of induction up to 72 weeks post-induction, at which time the animals were taken down for terminal cardiac assessment. The vehicle-treated *Pkp2-cKO* animals reached the humane endpoint by 6 weeks post-induction. There was no concerning decline in body weight for the treatment groups (all animals, including the ones that were found dead or euthanized were included). HBSS served as vehicle control. Error bar: s.e.m.. Sample size n=9, 10, 10, 11, 10, 11 for WT, cKO, cKO+AAV9:mPkp2 at three weeks before, right after, or 1 weeks after induction, and cKO+TN-401 at three weeks before, respectively.

Supplementary Figure 6: AAV9:mPkp2 showed dose-dependent efficacy in preserving ejection fraction and right ventricle size, reducing arrhythmia scores, and improved lifespan relative to vehicle-treated *Pkp2-cKO* Animals.

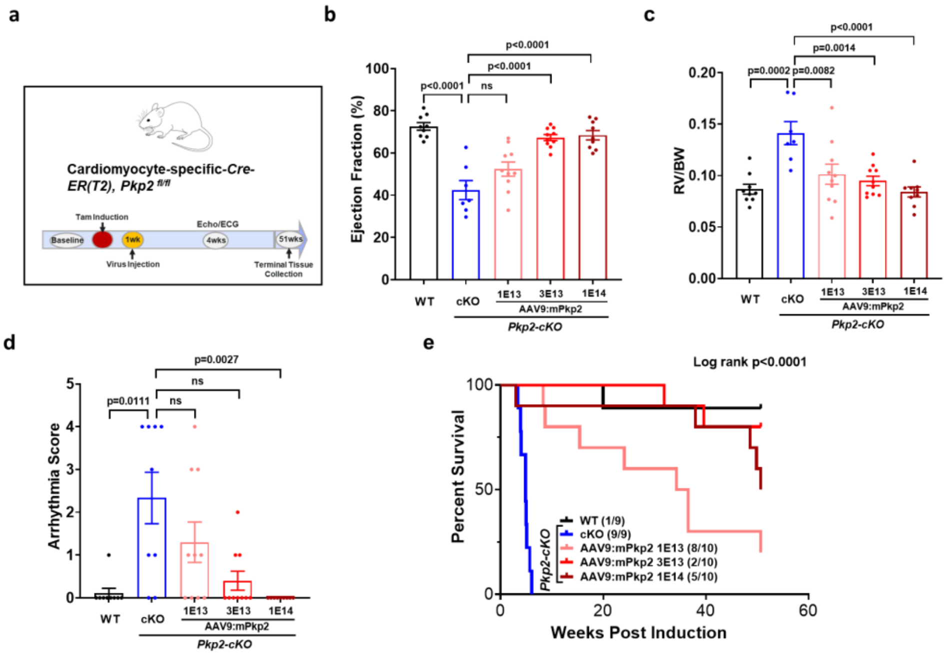

a, Study design describing animal model, virus injections, and timepoints for major functional readouts. AAV9:mPkp2 was injected one week after gene deletion at dose range of 1E13, 3E13, and 1E14 vg/kg. Echocardiograph (Echo) and electrocardiogram (EKG) data were collected at 4 weeks post gene deletion. **b,** EF% at 4 weeks post gene deletion. RV chamber dilation was measured by RV area normalized to body weight. **c,** RV/BW at 4 weeks post-induction. EF% and RV dilation in response to AAV9:mPkp2 treatment were statistically evaluated with ordinary One-Way ANOVA (Tukey's post-hoc test). Incidence of ventricular arrhythmia was quantified during 30 minutes of recording of anesthetized animals. The frequency and severity of the spontaneous arrhythmia were recorded and categorized based on the grading chart in supplementary Table 1. **d,** Ventricular arrhythmia score distribution of individual animals at 4 weeks post gene deletion. Statistical significance in response to AAV9:mPkp2 treatment was evaluated using Kruskal-Wallis test with Dunn's correction. **e,** Kaplan–Meier survival curve showed that AAV9:mPkp2 treatment extended life span of *Pkp2-cKO* mice with median lifespan to 35 weeks at 1E13 vg/kg and ≥ 50 weeks for both 3E13 and 1E14 vg/kg. Error bar: s.e.m.. Sample size n=9, 9, 10, 10, 10 for WT, cKO, cKO+AAV9:mPkp2 at 1E13, 3E13, and 1E14 vg/kg, respectively.

Supplementary Figure 7: TN-401 showed dose-dependent expression of human *PKP2* transgene protein and dose-dependent restoration of desmosome proteins.

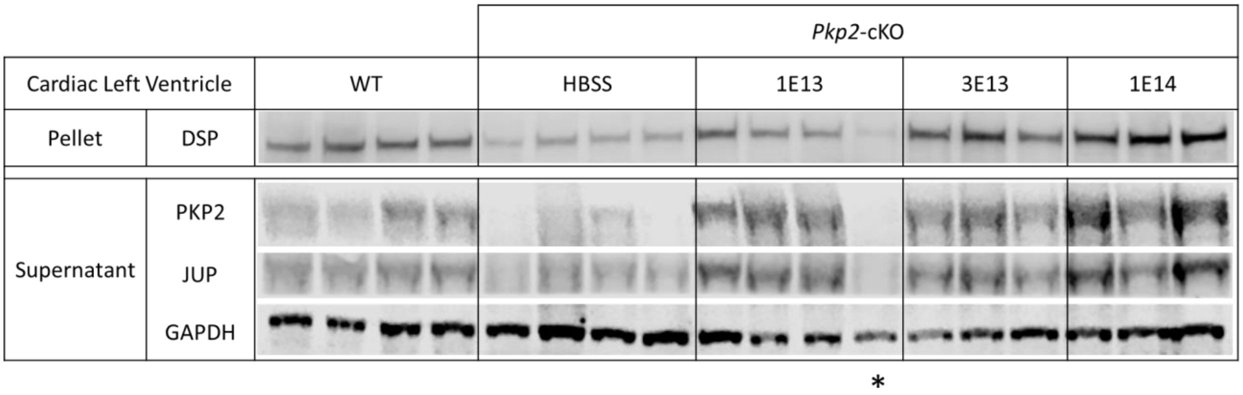

Dose-dependent efficacy of TN-401 was evaluated in *Pkp2-cKO* mouse model. Mice were injected with TN-401 at 1E13, 3E13, or 1E14 vg/kg at one week after tamoxifen induction of cardiac *Pkp2* gene deletion. At 4 weeks post tamoxifen induction (3 weeks post AAV9 injection), animals were sacrificed for expression and histological evaluation. Immunoblot analysis showed a dose-dependent expression of human PKP2 protein and restoration of endogenous JUP and DSP protein in LV. Below the graphs, raw immunoblot images were shown and * indicates animal found dead before protein analysis.

Supplementary Figure 8: TN-401 showed dose-dependent expression of human *PKP2* transgene protein.

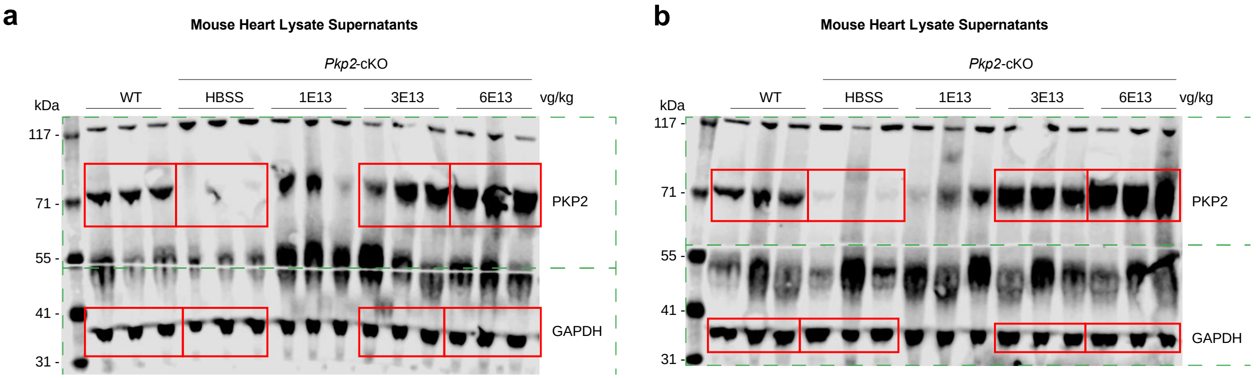

Representative Western blots for main Figure 7b. TN-401 dose-dependent expression of human PKP2 transgene protein was evaluated in *Pkp2-cKO* mouse model. Mice were injected with TN-401, produced from HEK293T, at 1E13, 3E13, or 6E13 vg/kg at one week before tamoxifen induction of cardiac *Pkp2* gene deletion. **a,** Mouse heart lysates (supernatant fraction) from three biological replicates show reduced levels of endogenous mouse PKP2 expression in HBSS (vehicle) treated *Pkp2-cKO* mice. TN-401 treatment resulted in expression of human PKP2 transgene protein in a dose dependent manner. GAPDH is probed as a loading control. **b**, Two additional biological replicates and one technical replicate from the first blot were run in the separate gel to accommodate comparison among three doses. Green boxes represent where blots are cut into segments to probe for proteins of interest. Red boxes highlight the bands quantified using Li-COR Image Studio densitometry. The 1E13 vg/kg dose was not quantified in the corresponding bar graph and is therefore not highlighted here.

Supplementary Table 1: Arrhythmia grade chart*

Grade	Arrhythmias
5	S-VT/VF/Cardiac Sudden Death
4	NSVT
3	>100 PVCs, couplets and triplets
2	>50, <100 PVCs
1	<50 PVCs, PJCs, AV block
0	<10 PVCs

* The frequency and severity of the spontaneous arrhythmia, including premature ventricular contractions (PVC), premature junctional complexes (PJC), atrioventricular (AV) block, non-sustained ventricular tachycardia (NSVT), sustained ventricular tachycardia (S-VT), ventricular fibrillation (VF) were recorded and evaluated for an overall composite arrhythmia score.
