# Supplementary material for: AAV9:PKP2 improves heart function and survival in a Pkp2-deficient mouse model of arrhythmogenic right ventricular cardiomyopathy
Source: Commun Med (Lond). 2024 Mar 18;4:38. doi: 10.1038/s43856-024-00450-w (PMC10948840; doi:10.1038/s43856-024-00450-w)
Supplement: Supplementary file 3 — Description of Additional Supplementary Files [file 43856_2024_450_MOESM3_ESM.pdf]

Description of Additional Supplementary Files

File Name: Supplementary Data 1

Description: Source data for Figures 1-9, Supplementary Figures 5-6, animal age and gender, and WPRE RNA standards and primer and probe information.
