# Supplementary material for: AAV9:PKP2 improves heart function and survival in a Pkp2-deficient mouse model of arrhythmogenic right ventricular cardiomyopathy
Source: Commun Med (Lond). 2024 Mar 18;4:38. doi: 10.1038/s43856-024-00450-w (PMC10948840; doi:10.1038/s43856-024-00450-w)
Supplement: Supplementary file 5 — Reporting Summary [file 43856_2024_450_MOESM5_ESM.pdf]

nature portfolio

Corresponding author(s): COMMSMED-23-0786-A

Last updated by author(s): Jan 14, 2024

Reporting Summary

Nature Portfolio wishes to improve the reproducibility of the work that we publish. This form provides structure for consistency and transparency in reporting. For further information on Nature Portfolio policies, see our Editorial Policies and the Editorial Policy Checklist.

Statistics

For all statistical analyses, confirm that the following items are present in the figure legend, table legend, main text, or Methods section.

n/a	Confirmed	
☐	☒	The exact sample size (*n*) for each experimental group/condition, given as a discrete number and unit of measurement
☐	☒	A statement on whether measurements were taken from distinct samples or whether the same sample was measured repeatedly
☐	☒	The statistical test(s) used AND whether they are one- or two-sided<br>*Only common tests should be described solely by name; describe more complex techniques in the Methods section.*
☒	☐	A description of all covariates tested
☐	☒	A description of any assumptions or corrections, such as tests of normality and adjustment for multiple comparisons
☐	☒	A full description of the statistical parameters including central tendency (e.g. means) or other basic estimates (e.g. regression coefficient) AND variation (e.g. standard deviation) or associated estimates of uncertainty (e.g. confidence intervals)
☐	☒	For null hypothesis testing, the test statistic (e.g. *F*, *t*, *r*) with confidence intervals, effect sizes, degrees of freedom and *P* value noted<br>*Give P values as exact values whenever suitable.*
☒	☐	For Bayesian analysis, information on the choice of priors and Markov chain Monte Carlo settings
☒	☐	For hierarchical and complex designs, identification of the appropriate level for tests and full reporting of outcomes
☒	☐	Estimates of effect sizes (e.g. Cohen's *d*, Pearson's *r*), indicating how they were calculated

Our web collection on statistics for biologists contains articles on many of the points above.

Software and code

Policy information about availability of computer code

Data collection

1) RNAseq data collection:
The libraries were sequenced as 2x50 base pair paired-end reads using Illumina NovaSeq 6000 using V1.5 reagent kit on S1 flow cell with an average of 25.66 million reads per each read file (51.32M reads per sample).
2) Contraction of iPSC-CM was recorded in bright field by SONY SI8000 imaging system.
3) Axion Biosystems: Maestro Pro to acquire cardiomyocyte contraction and electrophysiological properties from 2D momolayer culture.
4) Leica DMi8 inverted microscope with LAS X3.4.2 Life Science Microscope Software.
5) Molecular Devices ImageXpress Micro Confocal High-Content Imaging System with MetaXpress 6 imaging analysis software.
6) Transthoracic echocardiography was performed using high resolution micro-imaging systems (Vevo 3100 Systems, Fujifilm VisualSonics) equipped with a 25-55 MHz linear array transducer for mouse heart images acquisition.
7) Two lead ECG (leads I and II) were recorded Power Lab; AD Instruments; LabChart 7 Pro software version.

Data analysis

1) Statistics:
The numbers of technical and biological replicates and animals for each experiment are indicated in the figure legends. Normality and lognormality tests were performed first to determine whether a dataset is normally distributed. Statistical analyses, ordinary One-Way ANOVA (Tukey's post-hoc test), ordinary Two-Way ANOVA (Tukey's post-hoc test), nonparametric Kruskal-Wallis test with Dunn's correction, and Student's t-test, were performed using GraphPad Prism 9. Significant differences were defined as $p < 0.05$. Error bars in all mouse studies represent SEM (Standard Error of the Mean). Error bars in all cell biology studies represent SD (Standard Deviation). Statistical tests for each individual experiment are provided in the figure legend.
2) RNAseq data analysis:
After adapter trimming by fastp (version 0.23.3), raw RNA-seq reads from mouse hearts in fastq format were aligned with Salmon (version 1.8.0) to the GENCODE (version M30, July 2022) reference transcript assembly (GRCm39 and Ensembl 107) using best practice parameters to

ensure mapping validity and reproducibility (--seqBias --gcBias --posBias --useVBOpt --rangeFactorizationBins 4 --validateMappings --mimicStrictBT2). Next, a script using R package tximport was used to generate an expression matrix normalized to transcripts per million (TPM). In this analysis, we only used genes detected in at least 10% of all samples. Protein-coding genes were determined using Ensembl release mus musculus annotations (GRCm39, July 2022) and extracted by biomaRt (version 2.52.0). Mitochondrial genes were also omitted, followed by renormalization to TPM. These gene expression values were then log2-transformed after addition of 1 as pseudo-count. Expression patterns of key genes associated with functions of interest were visualized across treatment groups with boxplots generated using the ggplot2 R package. Expression values from both left and right ventricles were included in the boxplots. Relative gene expression levels across groups and two ventricles are also presented in scaled values per gene in the heatmaps. Heatmaps were generated in R using ComplexHeatmap package.
For initial assessment and identifying presence of cluster patterns in the transcriptome, Principal Component Analysis (PCA) models were generated in R using the `prcomp` function from the stats package. The first two principal components were used to visualize group level differences across samples in a PCA plot generated using ggplot2 and ggfortify packages with the `autoplot` function. Differential gene expression analysis was then performed by comparing each two groups of interest using Welch's t-test on pseudo-log normalized TPM values. The obtained t statistics values were used to rank-order the genes for the downstream functional analyses. Volcano plots were then generated to visualize the top positive and negative differentially expressed genes (DEGs) using the ggplot2 R package. The top DEGs are the set of genes with the highest and lowest t-statistics values. To evaluate functional effects, we performed Gene Set Enrichment Analysis (GSEA) on the gene list pre-ranked by t-statistics obtained from differential gene expression analysis, using the clusterProfiler R package. GSEA assesses whether differences in expression of predefined gene sets between two phenotypes are concordant and statistically significant. Gene sets were obtained from positional, curated canonical pathways, transcription factor targets, Gene Ontology, cell type signatures and Hallmark collections in Human MSigDB (v2023.1.Hs). Upon performing GSEA, these gene sets were only considered statistically significant if the false discovery rate (Q value) was less than 0.25 as determined with multiple hypothesis testing correction using the BH-correction method. The normalized enrichment score, which reflects the degree to which a gene set is overrepresented in the ranked list and normalized for gene set size, was used to select significantly altered gene sets. Trends in normalized enrichment scores for some gene sets of interest were shown in heatmaps, which were generated in R using ComplexHeatmap.

3) Acquired cell contraction videos were analyzed by DANA Solutions Pulse analysis software (now Curi Bio).
4) Maestro Pro software modules: Cardiac modules and MEA Viability.
5) Immunofluorescence images taken on Leica DMi8 inverted microscope by LAS X3.4.2 Life Science Microscope Software.
6) Confocal images were analyzed and quantified by MetaXpress 6 imaging analysis software.
7) Echocardiography imagin data were analyzed by Vevo Lab ultrasound analysis software 5.6.1.
8) Raw ECG trace were analyzed using a digital acquisition and analysis system of LabChart 8. Arrhythmia numbers and types were detected, hand-counted, and scored by an ECG expert.

For manuscripts utilizing custom algorithms or software that are central to the research but not yet described in published literature, software must be made available to editors and reviewers. We strongly encourage code deposition in a community repository (e.g. GitHub). See the Nature Portfolio guidelines for submitting code & software for further information.

Data

Policy information about availability of data

All manuscripts must include a data availability statement. This statement should provide the following information, where applicable:

- Accession codes, unique identifiers, or web links for publicly available datasets
- A description of any restrictions on data availability
- For clinical datasets or third party data, please ensure that the statement adheres to our policy

All RNAseq data will be deposited and accession codes with corresponding links will be made available to the public. All pharmacology data that support the findings of this study are not openly available due to reasons of sensitivity and are available from the corresponding author upon reasonable request. Data are located in controlled access data storage at Tenaya Therapeutics.

Research involving human participants, their data, or biological material

Policy information about studies with human participants or human data. See also policy information about sex, gender (identity/presentation), and sexual orientation and race, ethnicity and racism.

Reporting on sex and gender	n/a
Reporting on race, ethnicity, or other socially relevant groupings	n/a
Population characteristics	n/a
Recruitment	n/a
Ethics oversight	n/a

Note that full information on the approval of the study protocol must also be provided in the manuscript.

Field-specific reporting

Please select the one below that is the best fit for your research. If you are not sure, read the appropriate sections before making your selection.

☒ Life sciences ☐ Behavioural & social sciences ☐ Ecological, evolutionary & environmental sciences

For a reference copy of the document with all sections, see nature.com/documents/nr-reporting-summary-flat.pdf

Life sciences study design

All studies must disclose on these points even when the disclosure is negative.

Sample size	For animal studies, ejection fraction was the primary outcome measure used to determine sample size. In addition, the animal sample size per treatment group was determined based on our in-house experience of conducting efficacy, dose-ranging, and long-term survival benefit studies using AAV9 as test article.
Data exclusions	Animal death was documented and plotted in Kaplan-Meier survival curves and body weight curves. Animals that were euthanized or died prior to the last echocardiography or ECG were excluded from the time progression curve of EF%, RV size, and arrhythmia scores. Animals found dead were excluded from tissue DNA, RNA, or protein analyses. If an animal found dead right after echo or ECG, it would be included in terminal tissue processes.
Replication	SOP was strictly followed. The reproducibility was ensured by using animals that were age-matched with evenly distributed body weight and sex. Tamoxifen induction and AAV injection were performed by one scientist and echocardiography and ECG were performed by another scientist for consistency. ECG raw traces were examined and double checked by two different scientists. AAV9 was produced and purified following the SOP and each lot titer was determined based on vector genomes. All attempts on replication were successful.
Randomization	Animals were randomized for either the vehicle control or the AAV9-treated based on body weight and sex that were evenly distributed among treatment groups.
Blinding	Scientists were blinded to group allocation during data collection and analysis.

Reporting for specific materials, systems and methods

We require information from authors about some types of materials, experimental systems and methods used in many studies. Here, indicate whether each material, system or method listed is relevant to your study. If you are not sure if a list item applies to your research, read the appropriate section before selecting a response.

Materials & experimental systems

n/a	Involved in the study	
☐	☒	Antibodies
☐	☒	Eukaryotic cell lines
☒	☐	Palaeontology and archaeology
☐	☒	Animals and other organisms
☒	☐	Clinical data
☒	☐	Dual use research of concern
☒	☐	Plants

Methods

n/a	Involved in the study	
☒	☐	ChIP-seq
☒	☐	Flow cytometry
☒	☐	MRI-based neuroimaging

Antibodies

Antibodies used	1) Immunofluorescence: antibodies against anti-PKP2 (Invitrogen, rabbit polyclonal PA5-53144); anti-DSP (Invitrogen, rabbit polyclonal 25318-1-AP; Sigma, mouse monoclonal 498 MABT1492); anti-JUP (Sigma, mouse monoclonal P8087).<br>2) Histology: anti-PKP2 and anti-Cx43 (Invitrogen rabbit polyclonal PA5-53144 and rabbit polyclonal 71-0700).<br>3) Western blot: mouse anti-PKP2, rabbit anti-DSP, mouse anti-JUP, mouse anti-Cx43, and mouse anti-GAPDH (Santa Cruz Biotech SC-393711, Invitrogen 25318-1-AP, Sigma P8087, Invitrogen 35-5000-3D8A5, and Invitrogen MA5-15738, respectively).
Validation	Based on the manufacturer's recommendation, antibodies were tested on human iPSC derived cardiomyocytes and mouse tissue or cell and tissue lysate depending on the purpose of the applications.

Eukaryotic cell lines

Policy information about cell lines and Sex and Gender in Research

Cell line source(s)	iCell Cardiomyocytes2 (FUJIFILM Cellular Dynamics)
Authentication	Authentication of each lots were provided by the manufacturer.

Mycoplasma contamination	Negative on Mycoplasma contamination.
Commonly misidentified lines (See ICLAC register)	n/a

Animals and other research organisms

Policy information about studies involving animals; ARRIVE guidelines recommended for reporting animal research, and Sex and Gender in Research

Laboratory animals	Pkp2-cKO (αMyHC-Cre-ER(T2)/Pkp2fl/fl) mouse line in the C57BL/6 background
Wild animals	n/a
Reporting on sex	Animals were evenly assigned based on sex to each treatment. There was no sex-based analysis nor sex-specific findings.
Field-collected samples	n/a
Ethics oversight	Animal studies were performed according to Tenaya Therapeutics' animal use guidelines. The animal protocols were approved by the Institutional Animal Care and Use Committee (IACUC number: 2020.007).<br>Animals were allowed to acclimate for at least 3 days following shipment. Animals were housed in Innovive racks and disposable cages. The cages are pre-filled with alpha-dri as bedding. Our enrichments have nestlets and Twist-n'Rich for single-housed mice.

Note that full information on the approval of the study protocol must also be provided in the manuscript.
